# CRL4B E3 ligase recruited by PRPF19 inhibits SARS-CoV-2 infection by targeting ORF6 for ubiquitin-dependent degradation

**DOI:** 10.1128/mbio.03071-23

**Published:** 2024-01-24

**Authors:** Linran Zhang, Pengfei Hao, Xiang Chen, Shuai Lv, Wenying Gao, Chang Li, Zhaolong Li, Wenyan Zhang

**Affiliations:** 1 Institute of Virology and AIDS Research, The First Hospital of Jilin University, Changchun, Jilin, China; 2 Research Unit of Key Technologies for Prevention and Control of Virus Zoonoses, Chinese Academy of Medical Sciences, Changchun Veterinary Research Institute, Chinese Academy of Agricultural Sciences, Changchun, Jilin, China; 3 Department of Infectious Diseases, Infectious Diseases and Pathogen Biology Center, Key Laboratory of Organ Regeneration and Transplantation of The Ministry of Education, The First Hospital of Jilin University, Changchun, China; University of Calgary, Calgary, Alberta, Canada

**Keywords:** SARS-CoV-2, ORF6, PRPF19, CRL4B E3 ligase, proteasomal degradation

## Abstract

The accessory protein ORF6 of the severe acute respiratory syndrome coronavirus 2 (SARS-CoV-2) is a key interferon (IFN) antagonist that strongly suppresses the production of primary IFN as well as the expression of IFN-stimulated genes. However, how host cells respond to ORF6 remains largely unknown. Our research of ORF6-binding proteins by pulldown revealed that E3 ligase components such as Cullin 4B (CUL4B), DDB1, and RBX1 are potential ORF6-interacting proteins. Further study found that the substrate recognition receptor PRPF19 interacts with CUL4B, DDB1, and RBX1 to form a CRL4B-based E3 ligase, which catalyzes ORF6 ubiquitination and subsequent degradation. Overexpression of PRPF19 promotes ORF6 degradation, releasing ORF6-mediated IFN inhibition, which inhibits SARS-CoV-2 replication. Moreover, we found that activation of CUL4B by the neddylation inducer etoposide alleviates lung lesions in a SARS-CoV-2 mouse infection model. Therefore, targeting ORF6 for degradation may be an effective therapeutic strategy against SARS-CoV-2 infection.

**IMPORTANCE:**

The cellular biological function of the ubiquitin-proteasome pathway as an important modulator for the regulation of many fundamental cellular processes has been greatly appreciated. The critical role of the ubiquitin-proteasome pathway in viral pathogenesis has become increasingly apparent. It is a powerful tool that host cells use to defend against viral infection. Some cellular proteins can function as restriction factors to limit viral infection by ubiquitin-dependent degradation. In this research, we identificated of CUL4B-DDB1-PRPF19 E3 Ubiquitin Ligase Complex can mediate proteasomal degradation of ORF6, leading to inhibition of viral replication. Moreover, the CUL4B activator etoposide alleviates disease development in a mouse infection model, suggesting that this agent or its derivatives may be used to treat infections caused by SARS-CoV-2. We believe that these results will be extremely useful for the scientific and clinic communities in their search for cues and preventive measures to combat the COVID-19 pandemic.

## INTRODUCTION

The genome of SARS-CoV-2 is approximately 29.9 kb and is predicted to harbor at least 14 open reading frames (ORFs). The 5′-proximal two-thirds of the genome encodes 16 nonstructural proteins (nsp1-16), making up the replicase. The 3′ one-third of the genome encodes four structural proteins (S [spike], E [envelope], M [membrane], and N [nucleocapsid]) and group-specific (accessory) proteins (ORF3a, ORF3b, ORF6, ORF7a, ORF7b, ORF8, ORF9a, ORF9b, ORF10). The host innate immune system is the first line of defense against viral infections. It is, thus, not surprising that SARS-CoV-2 can effectively suppress interferon (IFN) production in the early phase of infection, and infected cells produce only limited pro-inflammatory cytokines and chemokines (1, 2). This deficiency of IFN responses directly contributes to not only productive viral replication but also pathology associated with SARS-CoV-2 infection. Nsp13, nsp14, nsp15, and accessory proteins ORF6, ORF3b, ORF7a, ORF7b, ORF8, ORF9b, and ORF10 are considered the most potent IFN antagonists (3–5). Among these, ORF6 plays an important role in host immune evasion by robustly blocking IFN responses (3, 6–9). ORF6 is a protein of 57 amino acids, which localizes to membranes of the endoplasmic reticulum (ER), autophagosome, and lysosomal (10). ORF6 interacts with importin karyopherin alpha 2 (KPNA2), thus inhibiting nuclear translocation of the IFN regulatory factor 3 (IRF3) (9). ORF6 interacts with the Nup98 nucleopore complex via its C-terminus, thus inhibiting the nuclear export of IFN mRNA (6, 11). Such interactions also block STAT1 nucleus import and reduce the expression of its target genes (12–14).

Targeting key viral proteins for degradation is an effective mechanism of host defense, which can be achieved by proteases, autophagy, or the ubiquitin-proteasome pathway. Ubiquitination is catalyzed by a three-enzyme catalytic cascade in which the E3 enzyme dictates substrate specificity (15, 16). Among the three major classes of E3 ubiquitin ligases, the RING (Really Interesting New Gene) E3 family can be further classified into the Cullin-RING ligases (CRLs), anaphase-promoting complex, and the Skp1-Cullin-F-box protein complex. CRLs E3 ligases are assembled on a Cullin scaffold, binding a RING-box protein at its N terminus, an adaptor protein, and a substrate receptor at its carboxyl terminus (17, 18). The pre-mRNA processing factor 19 (PRPF19) is one such substrate recognition receptor for Cullin4B (CUL4B)-based E3 Ligases (19–21), which has been shown to promote ubiquitination and subsequent degradation of mutant ATXN3-polyQ protein, causing spinocerebellar ataxia type 3 (SCA3) cytotoxicity and neurodegeneration (22). PRPF19 also plays a role in limiting the replication of the porcine epidemic diarrhea virus (PEDV) by promoting selective autophagy-mediated degradation of the viral N protein (23). PRPF19 interacts with the NS1 protein of influenza A virus although the biological significance of such interactions is unclear (24).

In this study, we found that PRPF19 is an intrinsic antiviral protein that limits SARS-CoV-2 replication by promoting ubiquitination and subsequent degradation of viral ORF6.

## RESULTS

### ORF6 is degraded by the ubiquitin-proteasome pathway

**Fig 1:**
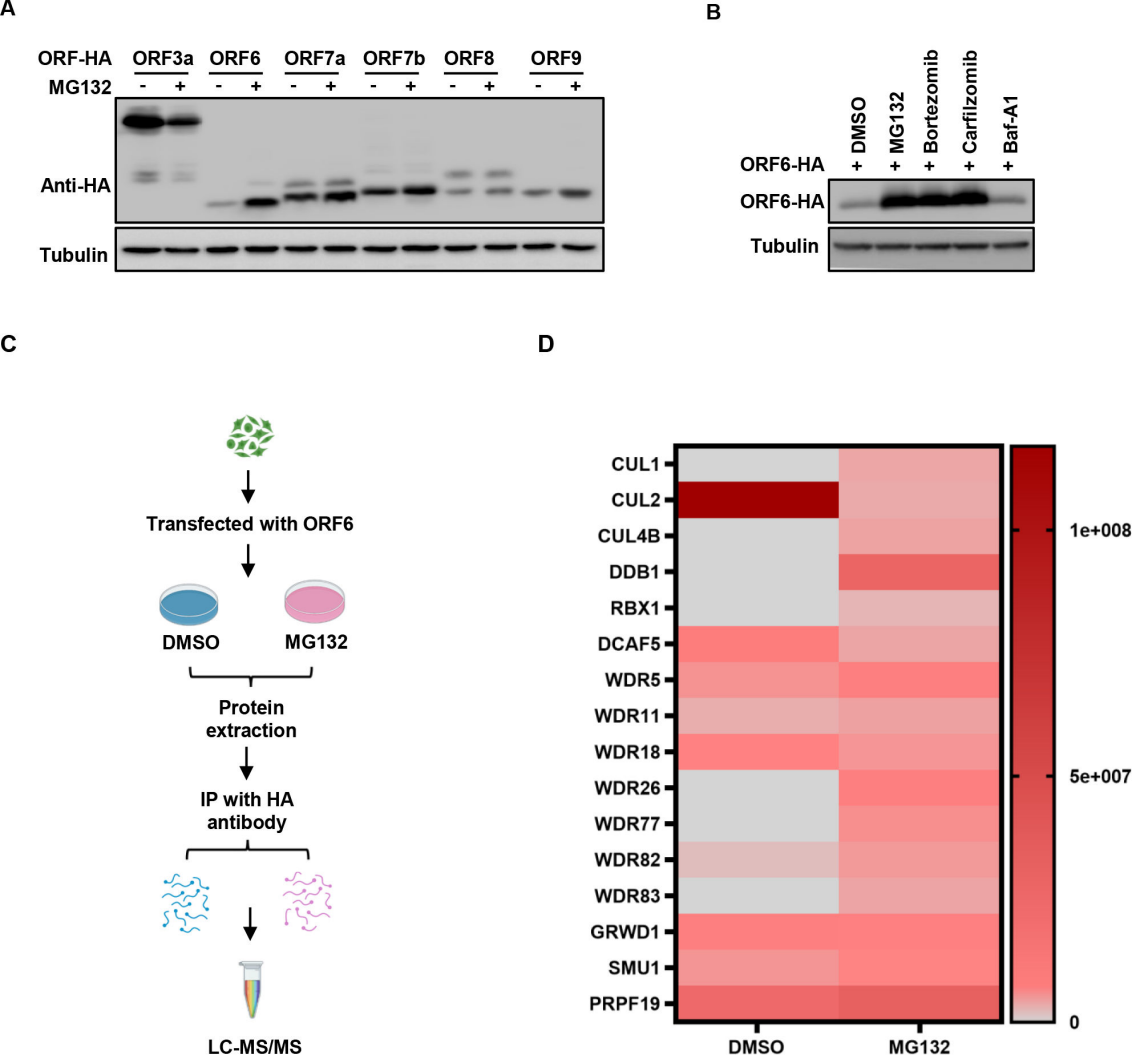
Identification of the ubiquitination-associated proteins involved in the degradation of SARS-CoV-2 ORF6. (**A**) The proteasomal inhibitor MG132 increased the stability of the ORF6 protein. HEK293T cells were transfected with SARS-CoV-2 accessory protein: ORF3a-HA, ORF6-HA, ORF7a-HA, ORF7b-HA, ORF8-HA, and ORF9-HA, and then, the cells were treated with or without 10 µM MG132 for 12 h prior to harvest. The cell lysates were analyzed by immunoblotting (IB). (**B**) Proteasomal inhibitors, but not other inhibitors, increased ORF6 stability. The ORF6-HA-tag expression vector was transfected into HEK293T cells, and then, the cells were treated with 10 µM MG132, Bortezomib, Carfilzomib, BafiloMycim AI, or DMSO for 12 h prior to harvest. The cell lysates were analyzed by IB. (**C**) Schematic of mass spectrometry experimental setup. (**D**) Binding Strength Analysis of the ubiquitination-associated proteins interacting with ORF6 in eluents.

To investigate whether the stability of nonstructural proteins of SARS-CoV-2 is regulated by the ubiquitin-proteasome pathway, we examined the effect of the proteasome inhibitor MG132 on the abundance of ORF3a, ORF6, ORF7a, ORF7b, ORF8, and ORF9 and found that MG132 treatment increased the protein levels of ORF6, ORF7a, ORF7b, and ORF9b but not ORF3a or ORF8 (Fig. 1A). Consistent with our recent study that ORF9b is degraded by the proteasome (25), while MG132 treatment increases its abundance. Because of ORF6’s role in anti-innate immunity, we further examined the role of the ubiquitin-proteasome pathway in the regulation of its stability and found that ORF6 stability is also sensitive to two other proteasome inhibitors, bortezomib, and carfilzomib but not the lysosomal inhibitor Bafilomycin A1 (Fig. 1B).

To identify the E3 ubiquitin ligase regulating ORF6 ubiquitination and subsequent proteasomal degradation, we treated HEK293T cells transfected with HA-ORF6 expression vector with MG132 and obtained potential binding proteins by immunoprecipitation (IP) with the HA-specific antibody (Fig. S1A). Mass spectrum and KEGG enrichment analysis revealed that ORF6 interacted with a variety of proteins associated with the proteasome degradation pathway (Fig. 1C; Fig. S1B). Importantly, compared to results obtained from control samples not treated with MG132, the abundance of a number of proteins, including CUL1, CUL4B, RBX1, DDB1, WDR5, WDR11, WDR26, WDR77, WDR82, WDR83, SMU1, and PRPF19, appears to be higher (Fig. 1D), suggesting that their potential roles in the degradation of the ORF6 protein.

### The CRL4B^PRPF19^ E3 ligase is responsible for ORF6 degradation

**Fig 2:**
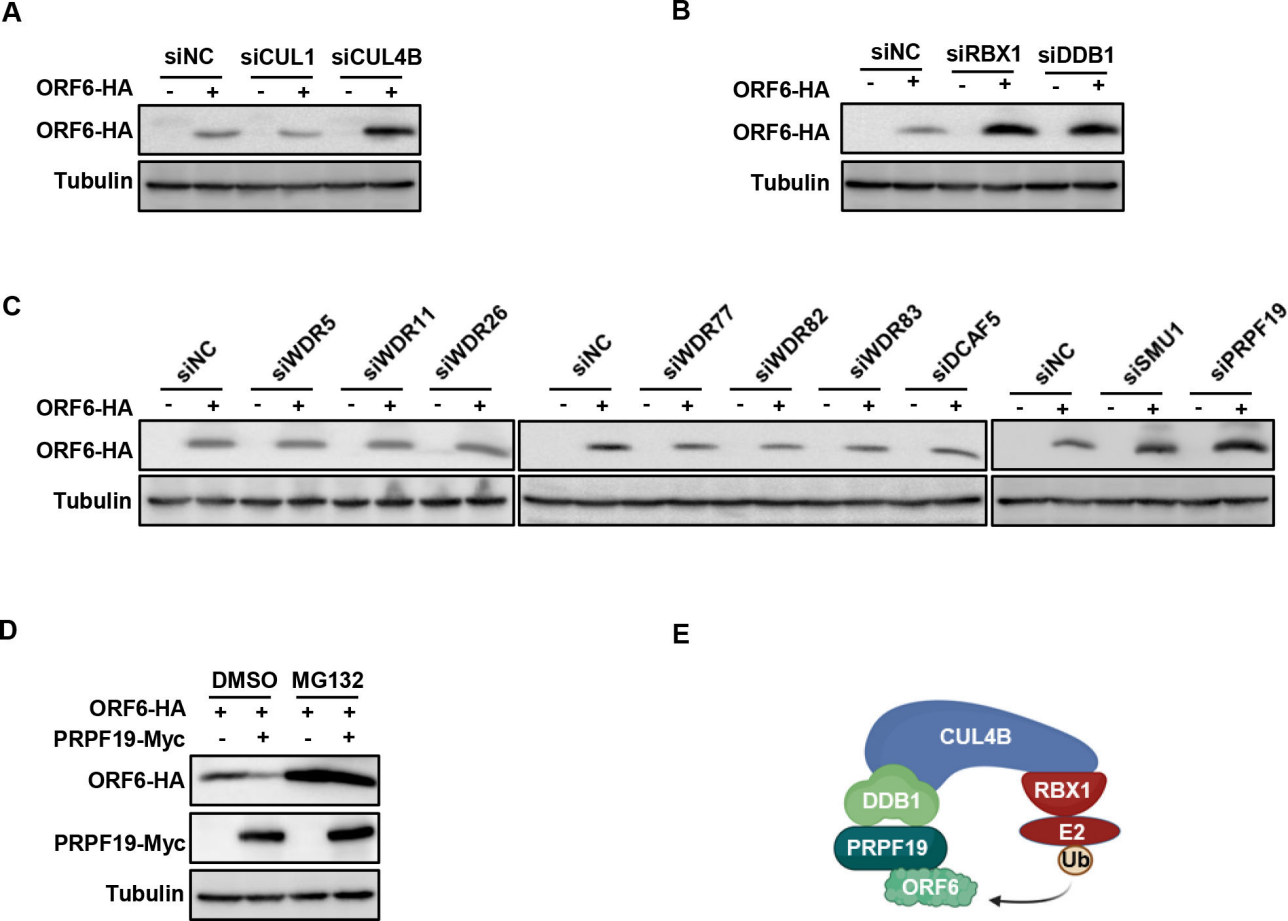
CRL4B^PRPF19^ E3 ligase targets ORF6 for degradation. (**A–C**) Knockdown of CUL4B, DDB1, RBX1, or PRPF19 increased ORF6 stability. Based on mass spectrum analysis results, CUL1, CUL4B, DDBI, RBXI, or the substrate recognition receptors (WDR5, WDR11, WDR26, WDR77, WDR82, WDR83, DCAF5, SMU1, PRPF19) were knocked down separately in HEK293T by siRNA, then the knockdown cells were transfected with ORF6-HA for 48 h. The cell lysates were analyzed by IB. (**D**) PRPF19 overexpression *in vivo* increased ORF6 degradation. ORF6 was cotransfected with a negative control vector or PRPF19 into HEK293T cells, and then, the cells were treated with or without 10 µM MG132 for 12 h prior to harvest. The cell lysates were analyzed by IB. (**E**) Schematic of CRL4B^PRPF19^-mediated ORF6 degradation. CUL4B formed a conserved E3 ligase complex with the RING-box protein RBX1, the adaptor protein DDB1, and the substrate recognition receptor PRPF19.

CRLs, the largest class of RING-type E3 ligases, utilize seven Cullins, including CUL1, CUL2, CUL3, CUL4A, CUL4B, CUL5, and CUL7 as scaffolds to assemble E3 ligase complexes for substrate ubiquitination (26, 27). Based on our mass spectrum analysis results, we first examined the importance of ORF6-interacting CUL1 and CUL4B in the ubiquitination and degradation of ORF6. The results showed that CUL1 knockdown had no impact on ORF6 stability, while knockdown of CUL4B significantly increased its abundance, suggesting its involvement in regulating ORF6 protein (Fig. 2A; Fig. S2A). CUL4B assembles CRL4 E3 ligases by forming protein complexes with the RING-box protein RBX1 and the adaptor protein DDB1, which were also identified in our pulldown experiments. As expected, knocking down RBX1 or DDB1 significantly increased ORF6 stability (Fig. 2B; Fig. S2B). To confirm that these proteins form an E3 complex that interacts with ORF6, we coexpressed ORF6-HA with CUL4B-Myc, RBX1-Flag, and DDB1-Flag and found that each of these proteins was present in precipitates obtained with immunoprecipitated with beads coated with the HA antibody (Fig. S2C). In contrast, none of these proteins was detected in precipitates from cells transfected to coexpress negative control vector VR1012. These results suggest that CUL4B, RBX1, and DDB1 form a CRL E3 complex targeting ORF6 for degradation.

In addition to the RING-box protein RBX1 and the adaptor protein DDB1, the formation of the CUL4B E3 ligase complex also needs substrate recognition receptors (DCAFs) (28). In the human genome, more than 100 substrate recognition receptors have been characterized according to the WD40 repeats that they contain (29, 30). Mass spectrometry data showed that nine DCAFs interacted with ORF6: WDR5, WDR11, WDR26, WDR77, WDR82, WDR83, DCAF5, SMU1, and PRPF19. To identify the specific DCAF regulating ORF6 degradation, we knocked down each of these DCAFs (Fig. S2D) and cotransfected with ORF6. As shown in Fig. 2C, only the knockdown of PRPF19 increased the stability of the ORF6 protein, whereas the knockdown of PRPF19 did not affect the stability of other viral proteins: E, S, M, N, ORF7a, ORF7b, ORF9 (Fig. S2E), indicating that PRPF19 specifically affects ORF6. Furthermore, overexpression of PRPF19 promoted the degradation of ORF6 (Fig. 2D). These results clearly demonstrated that CUL4B, RBX1, DDB1, and PRPF19 form a CRL4B^PRPF19^ E3 ligase and are responsible for ORF6 degradation (Fig. 2E).

### PRPF19 interacts with and catalyzes ORF6 protein via K48 ubiquitination

As the substrate recognition receptor, PRPF19 is able to connect substrate and protein complexes (31). The interaction between PRPF19 and substrate ORF6 was readily detectable in reciprocal co-immunoprecipitation (co-IP) experiments by using anti-Myc-agarose or anti-HA-agarose (Fig. S3A and B). Furthermore, fluorescence resonance energy transfer (FRET) analysis showed that the fluorescence signals of the ECFP-PRPF19 fusion became brighter after bleaching the signals of ORF6-YFP (Fig. S3C). These results suggested that PRPF19 interacts with ORF6 protein.

**Fig 3:**
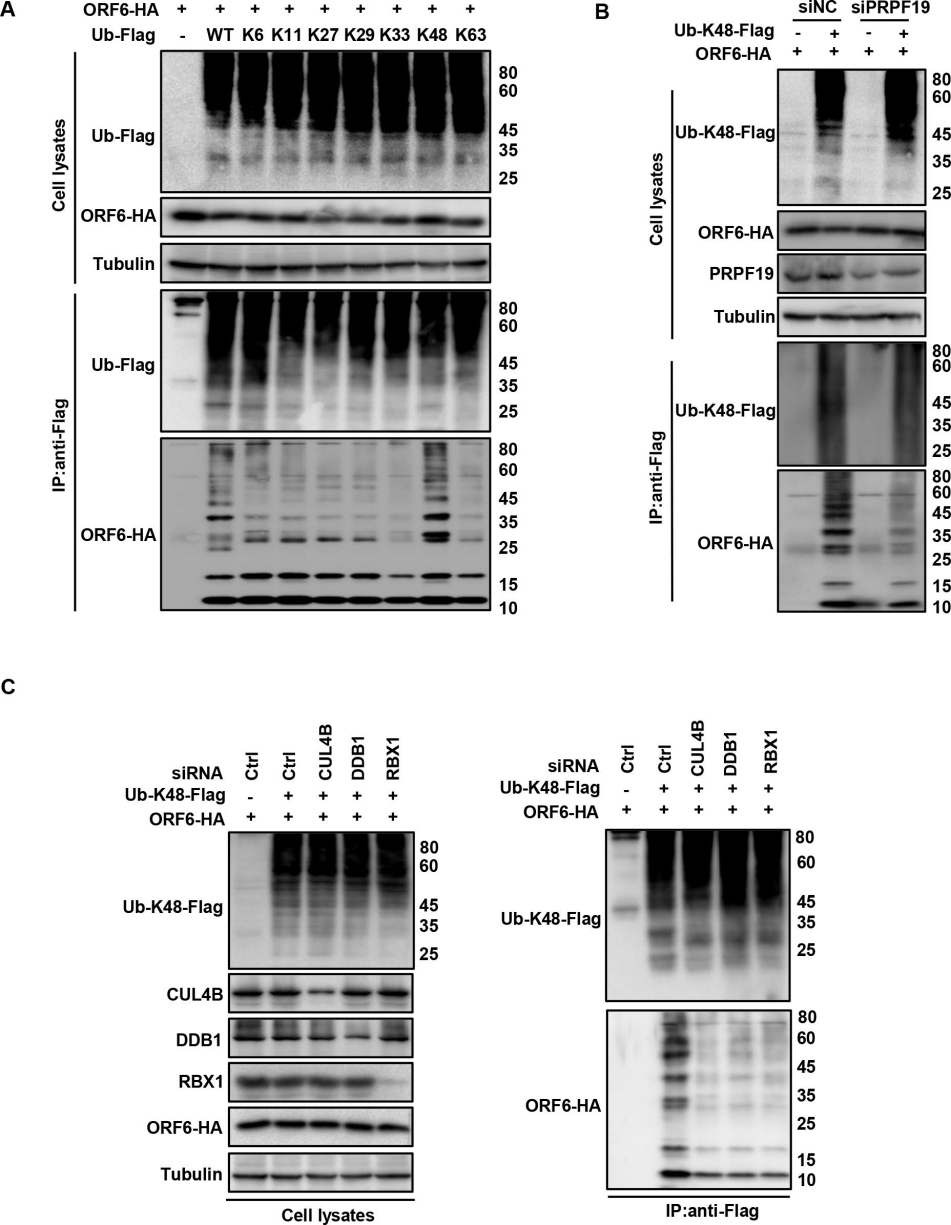
CRL4B^PRPF19^ catalyzes the formation of K-48 ubiquitin chains of ORF6. (**A**) ORF6 was ubiquitinated via K48-linked but not K6, K11, K27, K29, K33, or K63. HEK293T cells transfected with ORF6 and Ub-WT or Ub-mutants were treated with 10 µM MG132 for 12 h prior to harvest. Cell lysates were immunoprecipitated by protein G agarose beads conjugated with anti-Flag antibodies. Cell lysates and precipitated samples were analyzed by IB. (**B**) Knockdown of PRPF19 decreased K48-linked ubiquitination of ORF6. Lysates of PRPF19-knockdown HEK293T cells transfected to express ORF6-HA and ubiquitin-K48-Flag were subjected to Flag IP and then analyzed by IB. (**C**) Knockdown of CUL4B, DDB1, and RBX1 decreased K48-linked ubiquitination of ORF6. Lysates of CUL4B, DDB1, and RBX1 knockdown HEK293T cells transfected to express ORF6-HA and ubiquitin-K48-Flag were subjected to Flag IP and then analyzed by IB.

Polyubiquitination can occur in one of the seven lysine residues of ubiquitin, resulting in the formation of different types of ubiquitin chains, which dictate the fate of the modified protein (32, 33). To investigate the type of polyubiquitin chain formed on the ORF6 protein by PRPF19, we set up reactions with a series of ubiquitin mutants, each containing only one single lysine residue (-K6, -K11, -K27, -K29, -K33, -K48, K63) and detected the ubiquitination level by immunoblotting against ORF6. Our results showed that ubiquitination of the ORF6 protein could only be detected in reactions receiving the K48-only ubiquitin mutant (Fig. 3A). Furthermore, we examined the effect of PRPF19 or CUL4B or DDB1 or RBX1 on polyubiquitination of ORF6-HA with or without Ub-K48-Flag by its specific siRNA and found that the silencing of any component of E3 ligase complex all significantly reduced K48-linked ubiquitination of ORF6 (Fig. 3B and C). These data suggested that CRL4B^PRPF19^ E3 ligase contributes to ORF6 ubiquitination.

### Identification of the ubiquitination sites in ORF6

**Fig 4:**
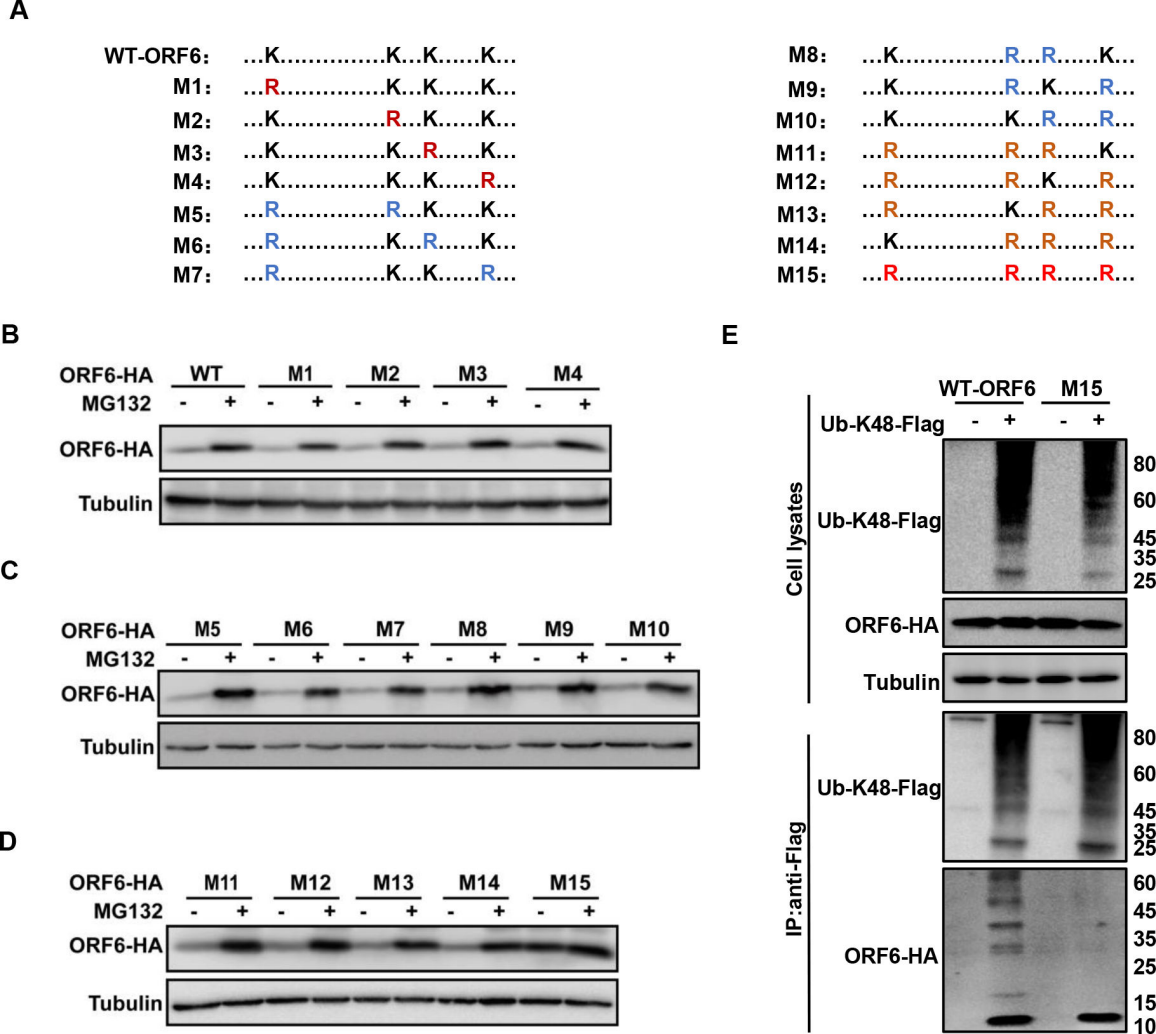
The ubiquitinated sites of ORF6. (**A**) Schema showing the lysines (**K**) mutants of SARS-CoV-2 ORF6. (**B–D**) The stability of ORF6 lysine mutants. HEK293T cells transfected ORF6-HA or ORF6 harboring lysine mutants were treated with 10 µM MG132 for 12 h prior to harvest. Cells were lyzed, and the level of relevant proteins was detected by IB. (**E**) The ubiquitination of ORF6 lysine mutants. HEK293T cells transfected with ORF6 WT or mutants plus K48-ubiquitin-Flag were treated with 10 µM MG132 for 12 h prior to harvest. Cell lysates were immunoprecipitated by protein G agarose beads conjugated with anti-Flag antibodies. Cell lysates and precipitated samples were analyzed by IB.

To identify the ubiquitination sites in ORF6 induced by PRPF19, we analyzed the full length of ORF6 and found four lysine sites, which were subsequently substituted by arginine separately or in combination. We examined the stability of ORF6 mutants with or without MG132 treatment and observed that only the ORF6-M15 was not degraded, indicating that four lysine residues are all required for ubiquitin degradation (Fig. 4A through D). Furthermore, we detected the degree of ubiquitination of the ORF6-M15. Consistent with our hypothesis, ORF6-M15 could no longer be ubiquitination modification (Fig. 4E), suggesting that K23, K38, K42, and K48 are all ubiquitination sites.

### PRPF19 antagonizes ORF6-mediated IFN signaling inhibition

**Fig 5:**
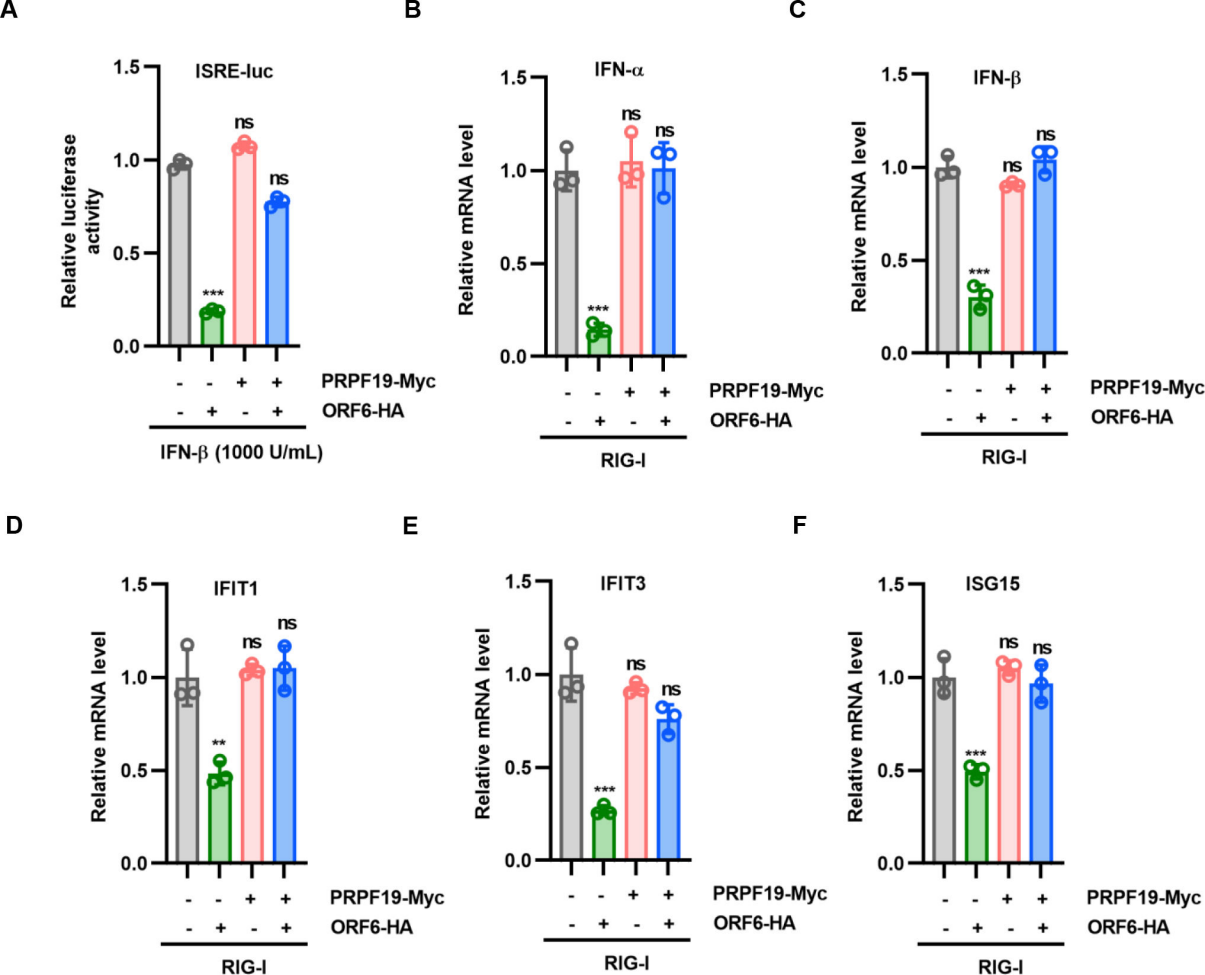
PRPF19 antagonizes ORF6-mediated IFN signaling inhibition by targeting the ORF6 protein for degradation. (**A**) HEK293T cells were transiently transfected with plasmids expressing ORF6-HA and PRPF19-Myc, a plasmid encoding ISRE-firefly luciferase reporter, as indicated. At 24 h Posttransfection, cells were treated with IFN-β (1,000 U/mL) for 8 h prior to measuring luciferase activities. (**B through F**) ORF6 plus negative control vector or PRPF19 were cotransfected with RIG-I(N) into HEK293T cells as indicated. Cells were harvested at 48 h post-transfection for RT-PCR. Data are representative of three independent experiments and shown as average ±SD (*n* = 3). Significance was determined by one-way ANOVA, followed by a Tukey multiple comparisons posttest. ns *P* > 0.05; **P < 0.01; ***P < 0.001.

Recent studies reported that ORF6 is the key IFN antagonist of SARS-CoV-2. The loss of ORF6 rendered the virus IFN-stimulating (3, 7, 12). It is, therefore, worthwhile to examine the effect of PRPF19 on ORF6-mediated IFN signaling inhibition. HEK293T cells were transfected with an ISRE-firefly luciferase reporter plasmid, ORF6, with or without PRPF19. IFN-β was used as the potent inducer for IFN production. The degree of IFN induction and suppression was assessed by quantitation of promoter activity of the IFN-stimulated response element. Consistent with other studies, we showed that ORF6 overexpression suppressed IFN-dependent ISRE induction. However, overexpression of PRPF19 induced the degradation of ORF6, resulting in ISRE releasing and loss of IFN-antagonizing (Fig. 5A). Furthermore, we determined the mRNA levels of IFN-α, IFN-β, and IFN-stimulated genes (ISGs) IFIT1, IFIT3, and ISG15 when ORF6 or ORF6 and PRPF19 were cotransfected with RIG-I(N) into HEK293T cells, as indicated. Similarly, PRPF19 enhanced ORF6 degradation, further upregulating the mRNA levels of IFN-α, IFN-β, and ISGs compared with ORF6 alone (Fig. 5B through F). These results indicate that PRPF19 could antagonize ORF6-mediated IFN signaling inhibition by mediating ORF6 degradation.

### PRPF19 antagonizes SARS-CoV-2 replication

**Fig 6:**
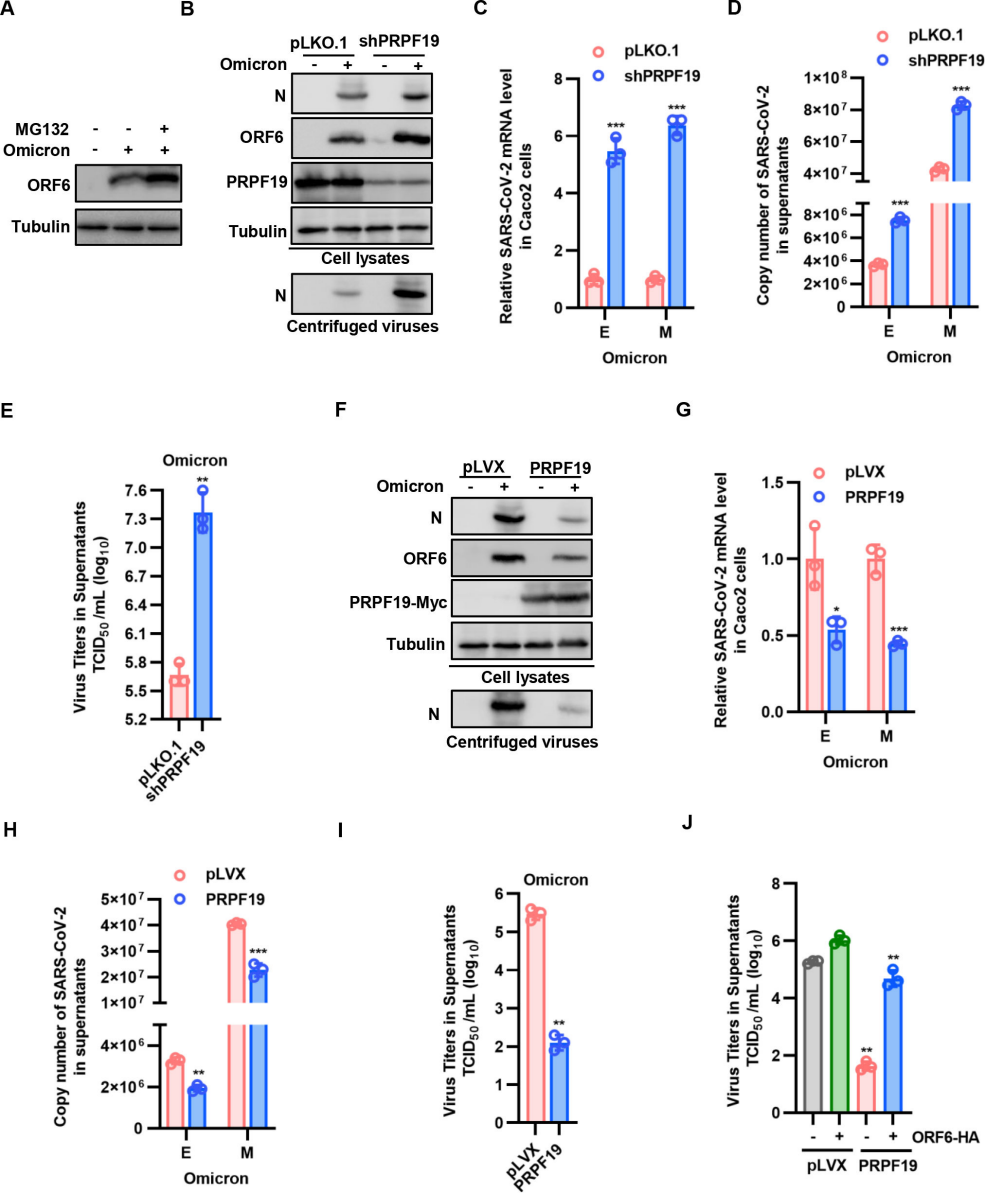
PRPF19 inhibits Omicron strain replication. (**A**) The proteasomal inhibitor MG132 increased the stability of the ORF6 protein in Omicron infection. PRPF19 knockdown in Caco2 cells increased Omicron strain replication measured by determining the protein levels of ORF6 and N (**B**), the mRNA levels of *E* and *M* genes in cells (**C**), and in culture supernatants (**D**), the virus titer in supernatants (**E**). PRPF19 overexpression in CaCO_2_ cells inhibited Omicron strain replication measured by determining protein levels of ORF6 and N (**F**), the mRNA levels of *E* and *M* genes in cells (**G**), and in culture supernatants (**H**), the virus titer in supernatants (**I**). (**J**) CaCO_2_ cells with stable expression of PRPF19 were transfected with negative control vector VR1012 or ORF6. At 24 h posttransfection, cells were infected with the Omicron strain. After another 48 h, the virus titer in supernatants was detected. Data are representative of three independent experiments and shown as average ±SD (*n* = 3). Significance was determined by a two-tailed *t*-test: *P < 0.05; **P < 0.01; ***P < 0.001.

To investigate whether the stability of ORF6 is regulated by the ubiquitin-proteasome pathway in SARS-CoV-2 infection, we examined the effect of the proteasome inhibitor MG132 on the abundance of ORF6 in Omicron strain infection. As expected, MG132 treatment increased ORF6 abundance (Fig. 6A). To further investigate the effect of cellular PRPF19 on viral replication, wild-type (WT) and PRPF19 knockdown cells were infected with SARS-CoV-2 Omicron strain or Wuhan strain for 48 h, and viral replication was assessed by determining the level of viral proteins, the level of viral mRNA within cells or in culture supernatant and virus titer in supernatant. Knocking down PRPF19 in Caco2 cells led to an increase in the level of ORF6, N, E, and M proteins of the Omicron strain (Fig. 6B through E). In addition, similar results were found on the Wuhan strain (Fig. S4A through D), indicating that knockdown of endogenous PRPF19 promoted the SARS-CoV-2 replication. Conversely, overexpression of PRPF19 restricted the viral RNA replication and viral protein expression of both Omicron and Wuhan strains (Fig. 6F through I; Fig. S4E through H). Furthermore, the decreased virus titer was rescued by overexpression of ORF6-HA (Fig. 6J), indicating that PRPF19 antagonizes SARS-CoV-2 replication by promoting ORF6 degradation.

### Enhanced CUL4B activity by etoposide suppresses SARS-CoV-2 virulence

**Fig 7:**
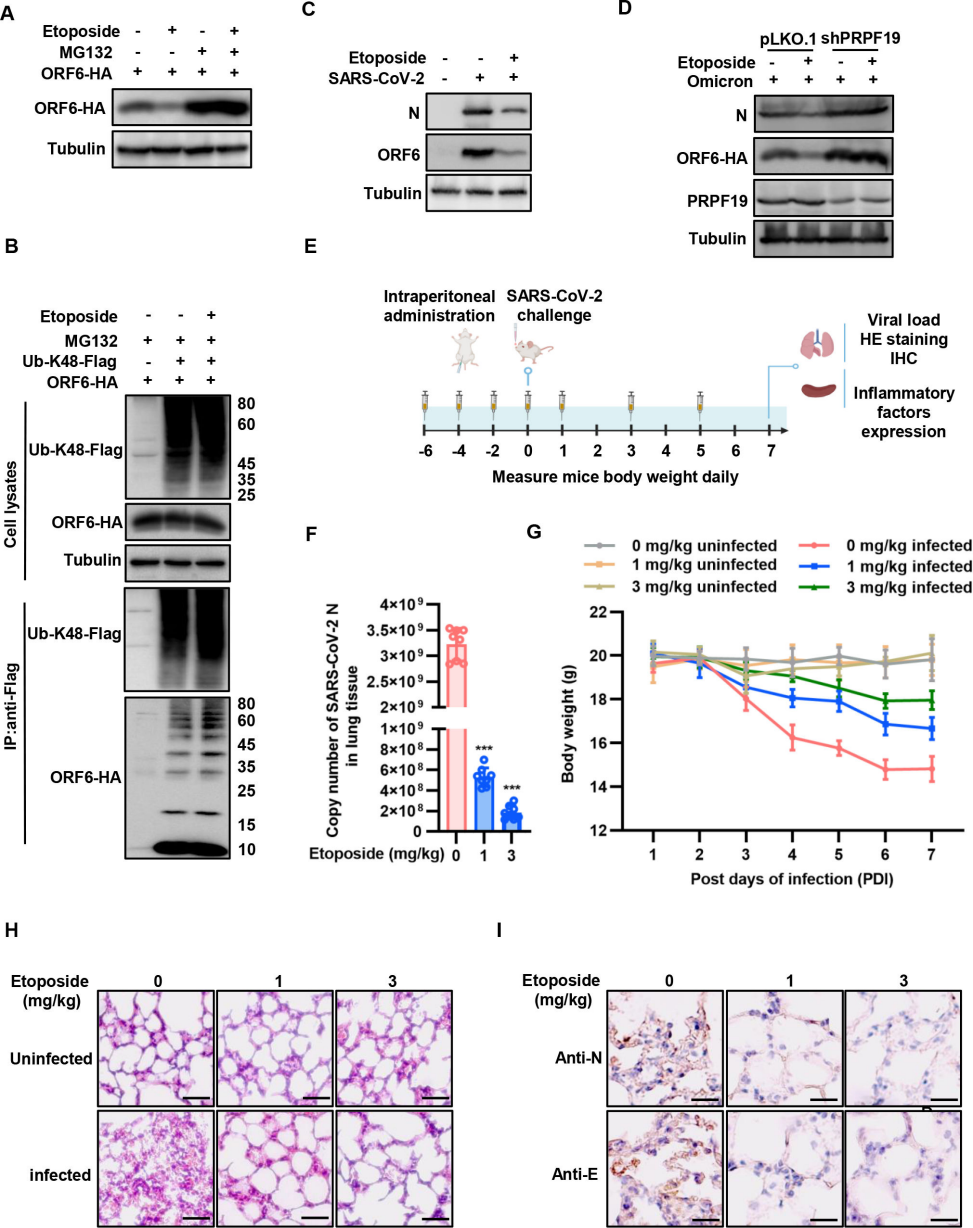
Activation of CUL4B increases ORF6 degradation and suppresses SARS-CoV-2 virulence. (**A**) Etoposide increased ORF6 degradation. HEK293T cells transfected with ORF6-HA were treated with 10 µM MG132 or 5 µM etoposide for 12 h prior to harvest. Cells were lysed, and the levels of relevant proteins were detected by IB. (**B**) Etoposide increased the ubiquitination level of ORF6. HEK293T cells transfected with ORF6-HA and K48-ubiquitin-Flag were treated with 10 µM MG132 and 5 µM etoposide for 12 h prior to harvest. Cell lysates were immunoprecipitated by protein G agarose beads conjugated with anti-Flag antibodies. Cell lysates and precipitated samples were analyzed by IB. (**C**) CaCO_2_ cells infected with SARS-CoV-2 were treated with or without etoposide (5 µM) for 24 h. Viral replication was determined by the protein levels of ORF6 and N. (**D**) CaCO_2_ cells with stable knockdown of PRPF19 were infected with SARS-CoV-2 and then treated with or without etoposide (5 µM) for 24 h. Viral replication was determined by the protein levels of ORF6 and N. (**E**) BLAB/C mice were treated with etoposide at a dosage of 0, 1, or 3 mg/kg (20 g mouse, 30 µg, or 60 µg) four times at 2-day intervals and then infected with the SARS-CoV-2 virus at a dosage of 10^5.5^ TCID50/mL via intraperitoneal injection. (**F**) Viral RNA loads of eight mice lungs were detected 7 days post-infection (dpi) by measuring the mRNA level of the *N* gene. (**G**) Weight of each mouse monitored over the experimental durations. (**H**) Representative images of H&E staining of lungs of differently treated mice. (**I**) The staining of viral N, M, and E proteins. Data are representative of three independent experiments and shown as average ±SD (*n* = 3). D and F significance were determined by a two-tailed *t*-test; G significance was determined by repeated measurement ANOVA. *P < 0.05; **P < 0.01; ***P < 0.001.

The activity of Cullins is regulated by neddylation. The cullin-linked Nedd8 can assist the neighboring protein in landing and positioning the E2 conjugating enzyme for the ubiquitin transfer reaction (34). Etoposide is a DNA damage inducer and a widely used chemotherapeutic agent for the treatment of various cancers (35). Recent studies showed that etoposide can induce CUL4B neddylation and promote the ubiquitination process (36). We, thus, examined the influence of etoposide on ORF6 ubiquitination and proteasomal degradation. The safe dose of etoposide was determined by CCK-8 assay (Fig. S5A and B). We observed that the etoposide (5 µM) treatment further reduced the cellular protein level of ORF6, while MG132 blocked the etoposide function (Fig. 7A). Treatment with etoposide also promoted the ubiquitination level of the ORF6 protein (Fig. 7B). To further examine the effect of etoposide on SARS-CoV-2 replication, the CaCO_2_ cells treated with or without etoposide (5 µM) for 24 h were infected with SARS-CoV-2. Etoposide treatment significantly reduced the protein levels of ORF6 and N compared to that of untreated samples (Fig. 7C). Similarly, the copies of the viral *N* gene in etoposide-treated cells were significantly lower (Fig. S5C). Importantly, etoposide lost antiviral function when PRPF19 was knocked down, suggesting that etoposide inhibits SARS-CoV-2 replication by regulating the CRL4B-PRPF19 complex (Fig. 7D).

We also determined the effect of etoposide on SARS-CoV-2 virulence in a mouse infection model. BALB/C mice were treated with one or 3 mg/kg etoposide by intraperitoneal injection four times at 2-day intervals and then infected with SARS-CoV-2 virus at a dosage of 10^5.5^ TCID50/mL (Fig. 7E). Compared to control groups, the copies of viral *N* gene in the lung of etoposide-treated mice were significantly lower (Fig. 7F). Furthermore, mice receiving the compounds exhibited less weight loss (Fig. 7G). Histopathological analysis of the major organs at 7 days post-infection (dpi) revealed that etoposide treatment alleviated lung lesions such as alveoli shrinkage and pulmonary edema typically observed in control mice (Fig. 7H). Consistently, etoposide treatment also reduced the abundance of N and E proteins (Fig. 7I). Together, these results indicate that etoposide inhibits SARS-CoV-2 virulence by promoting the degradation of the ORF6 protein.

A severe cytokine storm resulting from SARS-CoV-2 virus infection is thought to contribute to the high mortality rates (37). We also observed that mice infected with SARS-CoV-2 produced high levels of cytokines such as IL-1β, IL-6, and IL-1RA in the spleen. Importantly, etoposide treatment significantly reduced the production of these chemokines (Fig. S5D through F). In addition, many studies demonstrate that SARS-CoV-2 has evolved mechanisms to suppress innate immunity and steadily increase viral load. Our disease model also showed that mice infected with SARS-CoV-2 have a profoundly low production of IFNs at 7 dpi. Etoposide treatment reversed IFN-antagonizing and enhanced the antiviral effect (Fig. S5G through I).

## DISCUSSION

The emergence of SARS-CoV-2 has led to unprecedented medical and socioeconomic crises. Understanding the complicated virus-host interplay is helpful in developing strategies to control viral infection. Recent studies have demonstrated that ubiquitination and deubiquitination both regulate the replication of SARS-CoV-2 by modifying viral proteins or host factors. As the largest family of E3 ligases, RING E3s, the membrane-associated RING-CH 8 (MARCH8) and RING finger 5 (RNF5) were identified to induce the ubiquitination of SARS-CoV-2 spike or membrane proteins for lysosome degradation or the enhancement of virion release (38, 39). Our recent study identified that RNF5 also leads to the ubiquitination and degradation of the SARS-CoV-2 envelope, thereby inhibiting viral replication (40). The regulation of deubiquitinases (DUBs) on SARS-CoV-2 infection has also been investigated, such as DUB USP10, which is important for B cell response to SARS-CoV-2 or HIV-1 nanoparticle vaccines through deubiquitinating AID (41). Our recent study discovered that USP29 protects ORF9b from proteasome degradation by reversing ORF9b ubiquitination (25). Nevertheless, more details of the underlying regulatory mechanism of ubiquitination during SARS-CoV-2 infection need to be further investigated.

RING-E3s exist as monomers, dimers, and in complex multi-subunit assemblies. Monomers MARCH8 and RNF5, the CRL-based complex has been determined to play key roles in SARS-CoV-2 infection (34, 42). SARS-CoV-2 infection causes loss of smell and taste, which might be due to the fact that ORF10 hijacks CUL2ZYG11B to eliminate intraflagellar transport (IFT) complex B protein 46 (IFT46) and leads to cilia dysfunction (43). Actually, the phenomenon that various viral proteins usurp the CRL complex has been demonstrated as a classical model previously. For example, HIV-1 Vif recruits CUL5-ElonginB-ElonginC-CBFβ (CRL5) to antagonize host defensive factor APOBEC3G (A3G) (44). HIV-2 Vpx, simian virus 5 V (SV5-V), and hepatitis B virus X (HBx) proteins all hijack CRL4-DDB1 to induce the degradation of SMAHD1 (SAM domain and HD domain-containing protein 1), STAT1, or the cellular structural maintenance of chromosomes 5/6 complex, respectively (45–47). On the other hand, the CRL complex is also utilized by the host’s innate immunity to antagonize the virus (48). Here, we demonstrate that substrate recognition receptor PRPF19 interacts with CUL4B, DDB1, and RBX1 to form an E3 ligase, followed by ORF6 ubiquitination and degradation. PRPF19 has been demonstrated to limit PEDV replication (23) although antiviral mechanism is different from other host antiviral factors (49).

Discovering the mechanism of how CRL4-DDB1 E3 ligase regulates the biological process will provide a potential novel target for disease control. Small molecule TSC01682 disrupting the CUL4B-DDB1 interaction inhibits osteosarcoma cell growth (50). The activity of CRLs is regulated through neddylation, and DNA damage can induce neddylation (45). Previous study has demonstrated that DNA damage inducer etoposide can induce CUL4B neddylation, then enhancing its activity (36). Therefore, we treated cells or mice with different concentrations of etoposide before SARS-CoV-2 infection or challenge and found that etoposide effectively inhibits SARS-CoV-2 yield at the cell level (Fig. 7B and C) and reduces the copies of viral *N* gene in lung tissue, alleviates lung lesions such as alveoli shrinkage and pulmonary edema in the SARS-CoV-2-infected mice (Fig. 7D through H). Thus, the compound etoposide may be investigated to be a candidate for SARS-CoV-2 infection in the future.

In summary, PRPF19, as a substrate recognition receptor of E3 ligase, interacts with host factors CUL4B, DDB1, and RBX1 and forms a CRL4B-based E3 ligase to induce the ubiquitination and degradation of ORF6, thereby affecting SARS-CoV-2 infection (Fig. S7). Our study discovered that the molecular mechanism governing SARS-CoV-2 pathogenesis is helpful in designing a better therapeutic strategy.

## MATERIALS AND METHODS

### Plasmid construction

The DNAs encoding for ORF3a-HA, ORF6-HA, ORF7a-HA, ORF7b-HA, ORF8-HA, and ORF9-HA were synthesized by Shanghai Generay Biotech Co., Ltd. (Shanghai, China). ORF6 mutants were generated from ORF6-HA by site-directed mutagenesis. Human ubiquitin protein and its mutants carrying an N terminal Flag tag were inserted into VR1012 as SalI/BamHI fragments. DDB1-Flag (51), RBX1-Flag (52), CUL4B-Myc (53), and ISRE-Luc (8) have been previously described. Human PRPF19 fragments carrying a Myc tag at the N terminal end were amplified from cDNAs of HEK293T cells and then inserted into SalI/BglII of VR1012. Truncations of PRPF19-Myc were constructed by PCR with primers listed in Table S1. For stable expression, PRPF19 WT was inserted into pLVX-puro (BD Biosciences Clontech, catalog no. 632164) as XhoI/XbaI fragments. The coding region of ORF6 of SARS-CoV-2 and human PRPF19 were inserted into pCDNA3-YFP (Addgene, catalog no. 13033) and pECFP-C1 (BD Biosciences Clontech, catalog no. 6076-1), respectively, for immunofluorescence (IF) and FRET assays.

### siRNA and shRNA construction

Chemically synthesized short interfering RNA (siRNA) and a nonspecific control were purchased from RiboBio Co. Ltd. (Guangzhou, China). PRPF19-specific shRNA with the following target site was cloned in the lentiviral vector pLKO.1-puro (Addgene, catalog no. 8453). shPRPF19: 5′- CCGGGAACGGATGTGGAAGGAAGAACTCGAGTTCTTCCTTCCACATCCGTTCTTTTTG-3′ and 5′- AATTCAAAAAGAACGGATGTGGAAGGAAGAACTCGAGTTCTTCCTTCCACATCCGTTC-3′.

### Construction of stably silenced and overexpression cell lines

HEK293T cells were cotransfected with sh-PRPF19-pLKO.1 or pLKO.1 plus RRE, REV, and VSV-G expression vectors by using Lipofectamine 2000 (Invitrogen, Carlsbad, CA, USA). Packed lentiviral particles collected 48 h after transfection were used to infect Caco2 cells for 48 h, and puromycin (3 µg/mL, Sigma, St. Louis, MO, USA) was then added into the culture to select for stable cell lines. For stable overexpressing cell lines, pLVX-PRPF19 or pLVX was introduced, and the Vero-E6 cell lines were similarly selected and screened by 3 µg/mL puromycin.

### Cell culture and viruses

HEK293T (American Type Culture Collection[ATCC], Manassas, VA, USA, catalog no. CRL-11268), Caco2 (ATCC catalog no. HTB-37), and Vero-E6 (ATCC, catalog no. CRL-1586) cells were cultured as monolayers in Dulbecco’s modified Eagle’s medium (DMEM) (Hyclone, Logan, UT, USA) supplemented with 10% heat-inactivated (56°C, 30 min) fetal calf serum (FCS, GIBCO BRL, Grand Island, NY, USA) and maintained at 37°C with 5% CO_2_ in a humidified atmosphere. SARS-CoV-2 Wuhan (WuhanCoV/Beijing/IME-BJ05-2020, GenBank access no. MT291831.1) or Omicron (human/CHN_CVRI-01/2022) viruses were propagated in Vero E6 cells in DMEM supplemented with 2% FBS and titered using the median tissue culture infectious dose (TCID_50_) assay. All experiments of infectious SARS-CoV-2 were conducted under Biosafety Level 3 facilities.

### Transfection and infection

DNA transfections were carried out by Lipofectamine 3000 Reagent (Invitrogen, catalog no. L3000-008) according to the manufacturer’s instructions. siRNA transfections were carried out by Lipofectamine RNAiMAX Reagent (Invitrogen, catalog no. 13778150).

For SARS-CoV-2 infection, cells grown to 70% confluence in 6-well plates were washed twice with phosphate-buffered saline (PBS) and incubated with the indicated viral strains at 37°C for 1 h at an MOI of 0.01. Plates were gently agitated at 15 min intervals to facilitate adsorption. After adsorption, the virus-containing medium was replaced with fresh medium containing 2% FCS, followed by incubation at 37°C in 5% CO_2_ for the indicated durations.

### Antibodies and immunoblotting

Transfected or infected HEK293T, CaCO^_2_^, or Vero-E6 cells were harvested and boiled in 1× loading buffer (0.08 M Tris, pH 6.8, with 2.0% SDS, 10% glycerol, 0.1 M dithiothreitol, and 0.2% bromophenol blue) followed by separation on a 12% polyacrylamide gel. Proteins were transferred onto a polyvinylidene fluoride membrane for IB analysis. The membranes were incubated with primary antibodies, followed by a corresponding horse radish peroxidase (HRP)-conjugated secondary antibody (Jackson Immunoresearch, West Grove, USA, catalog no. 115-035-062 for anti-mouse and 111-035-045 for anti-rabbit) diluted 1:10,000, respectively. Proteins incubated with HRP-conjugated secondary antibodies were visualized using the ultra-sensitive ECL chemiluminescence detection kit (Proteintech, Rosemont, IL, USA, catalog no. B500024).

The following antibodies were used in this study: PRPF19 polyclonal antibody (pAb) (Sangon Biotec, Shanghai, CHN, catalog no. D127544), SARS-CoV-2 nucleocapsid antibody (GeneTex, Irvine, CA, USA, catalog no. GTX635679), SARS-CoV-2 ORF6 antibody (FabGenni, catalog no. SARS-COV2-ORF6-101AP), anti-myc pAb (Proteintech, catalog no. 16286-1-AP), anti-hemagglutinin (anti-HA) pAb (Invitrogen, Carlsbad, USA, catalog no. 71-5500), anti-tubulin mAb (Abcam, Cambridge, Cambridgeshire, UK, catalog no. ab11323), anti-Flag mAb (Sigma, Saint Louis, USA, catalog no. F1804).

### RNA extraction and RT-qPCR

Viral or intracellular RNA was isolated with TRIzol reagent by following the manufacturer’s instructions (15596-026; Invitrogen, Carlsbad, CA, USA). The cDNA was generated by EasyScript’s first-strand cDNA synthesis supermix (AE301; TransGen Biotech, Beijing, China). A total of 1 µg RNA was used as a template for each cDNA synthesis reaction. cDNA was stored at −80°C until use. The quantitative real-time PCR (qPCR) was carried out on an Mx3005P instrument (Agilent Technologies, Stratagene, USA) using the Power SYBR green PCR master mix (2×) (4367659; ABI). Quantitative RT-PCR (qRT-PCR) amplification of the target fragment was carried out with initial activation at 95°C for 2 min, followed by 45 cycles at 95°C for 15 s, 57°C for 15 s, and 68°C for 20 s. All primers for qPCR were presented in Table S1.

### Co-immunoprecipitation assay

For IP of proteins with an HA tag, HEK293T cells were transfected with indicated plasmids for 48 h, then harvested and washed twice with cold PBS, followed by ultrasonication with a lysis buffer (PBS containing 1% Triton X-100) and complete protease inhibitor cocktail (Roche, Basel, Basel-City, Switzerland, catalog no. 11697498001) and 10 µM MG132 (Abcam, catalog no. ab141003) at 4°C for 1 h. Cell lysates were cleared by centrifugation at 10,000 × *g* for 30 min at 4°C. Anti-HA agarose beads (Roche, catalog no. 11867423001) were mixed with the pre-cleared cell lysates and incubated at 4°C for 4 h on an end-over-end rocker. The reaction mixtures were then washed six times with cold wash buffer (20 mM Tris-HCl, pH 7.5, 100 mM NaCl, 0.1 mM EDTA, 0.05% Tween-20) and subsequently analyzed by IB. For Ub-Flag and PRPF19-Myc IP, cell lysates were mixed with anti-Flag or anti-Myc antibodies and protein-G agarose beads (Roche, catalog no. 11243233001).

### CCK-8 assay

Cells were first counted, and approximately 4000 cells per well were seeded in a 96-well cell culture plate (Corning Inc.). Then, after incubation at 37°C in a humidified atmosphere with 5% CO_2_ for 24 h, the culture medium was replaced by a series of concentrations of etoposide for an extra 12 h. Finally, 10 µL of the CCK-8 reagent was added into each well, and OD at 450 nm was measured using a multifunction microplate reader after incubation for 2 h at 37°C.

### Mass spectrometry

HEK293T were transfected with SARS-CoV-2-ORF6-HA for 36 h and then treated with MG132 or DMSO for 12 h prior to harvest. Co-IP assay was performed with HA beads (Roche, catalog no. 11867423001), and the elution was analyzed by mass spectrum. Mass spectrum analysis were performed by the National Center for Protein Science (Beijing, CHN).

### Fluorescence resonance energy transfer analysis

Hela cells seeded in 6-well glass-bottom plates were transfected with ORF6-YFP (1 µg) and ECFP-PRPF19 (1 µg) and then were treated with 10 µM MG132 to avoid the degradation for 12 h prior to fixing, following fixing in 4% paraformaldehyde at room temperature for 15 min and washing with PBS for 3 times. Fluorescent images of samples were then acquired with an Olympus FV 3000 confocal imaging system. CFP was excited at 458 nm, and the emission was collected through a 470–500 nm bandpass filter. YFP was excited at 514 nm, and the emission was collected through a 525–575 nm filter. To study protein interactions, we selected ROIs and measured donor fluorescence intensity before the bleaching step. We performed bleaching with 100% laser power for the acceptor. Finally, we measured fluorescence intensity after acceptor photobleaching and calculated FRET efficiency.

### Luciferase assays

HEK293T cells were seeded in 24-well plates and transfected with ISRE-luc firefly luciferase reporter and Renilla luciferase reporter (Promega), together with the indicated combination of expression plasmids. At 24 h posttransfection, cells were transfected with plasmids encoding RIG-I for another 24 h or treated with IFNb for another 8 h. Luciferase activity was measured using the Dual-Luciferase Reporter Assay System (E1910; Promega, Madison, WI, USA) according to the manufacturer’s protocol using a GloMax 20/20 Luminometer (Promega).

### Mouse lines and infection

BALB/C mice were purchased from Charles River Laboratories (Beijing, CHN). All welfare and experimental procedures were carried out strictly in accordance with the Guide for the Care and Use of Laboratory Animals and the related ethical regulations. All efforts were made to minimize animal suffering. The mice were randomly divided into six groups, and each group contained eight mice. Three groups were treated with the dosage of 0, 1, 3 mg/kg etoposide for four times every 2 days via intraperitoneal injection, then infected with SARS-CoV-2 isolate at a dosage of 10^5.5^ TCID50/mL via intranasal challenge, etoposide was injected again on the first, third, and fifth day post-infection. No etoposide treatment and SARS-CoV-2 infection was used as a negative control. 1 and 3 mg/kg of etoposide treatment only was used to evaluate the toxicity of etoposide.

### Immunohistochemical analysis

A total of 18 mice (3 mice in each group) were anesthetized, lung and spleen were harvested. Lungs were fixed with 3.7% paraformaldehyde solution for 2 days. Then, all of the lungs were dehydrated via an ethanol gradient, clarified through dimethylbenzene, and embedded in paraffin, and 4 µm sections were obtained for hematoxylin and eosin (H&E) staining. Histopathological analysis of the lungs was performed under a light microscope. The endogenous peroxidase activity of the tissues was inhibited by treatment with hydrogen peroxide (2.5%). Amount of SARS-CoV-2 N and E proteins in lung were detected by SARS-CoV-2 nucleocapsid monoclonal antibody (mAb) (GeneTex, catalog no. GTX635679) and SARS-CoV-2 Envelope antibody (GeneTex, catalog no. GTX136046), and a Streptavidin-Peroxidase Anti-Rabbit IgG kit (Maixin, Fuzhou, CHN, catalog no. KIT-9706).

### Statistical analysis

The detailed statistical analysis has been described in figure legends. All data are expressed as the mean ± standard deviations (SDs). Statistical comparisons were made using a student’s *t*-test, one-way ANOVA, or repeated measurement ANOVA. Significant differences are indicated in figures as follows: *P < 0.05, **P < 0.01, and ***P < 0.001; ns stands for no significance.

## SUPPLEMENTAL MATERIAL

Supplemental material mbio.03071-23-s0001.docx Supplemental figures and tables.

ASM does not own the copyrights to Supplemental Material that may be linked to, or accessed through, an article. The authors have granted ASM a non-exclusive, world-wide license to publish the Supplemental Material files. Please contact the corresponding author directly for reuse.
