# Supplementary material for: CRL4B E3 ligase recruited by PRPF19 inhibits SARS-CoV-2 infection by targeting ORF6 for ubiquitin-dependent degradation
Source: mBio. 2024 Jan 24;15(2):e03071-23. doi: 10.1128/mbio.03071-23 (PMC10865787; doi:10.1128/mbio.03071-23)
Supplement: Supplemental material — Supplemental figures and tables. [file mbio.03071-23-s0001.docx]

**Supplemental Materials For**

**CRL4B E3 ligase recruited by PRPF19 inhibits SARS-CoV-2 infection by** **targeting ORF6 for** **ubiquitin-dependent** **degradation**


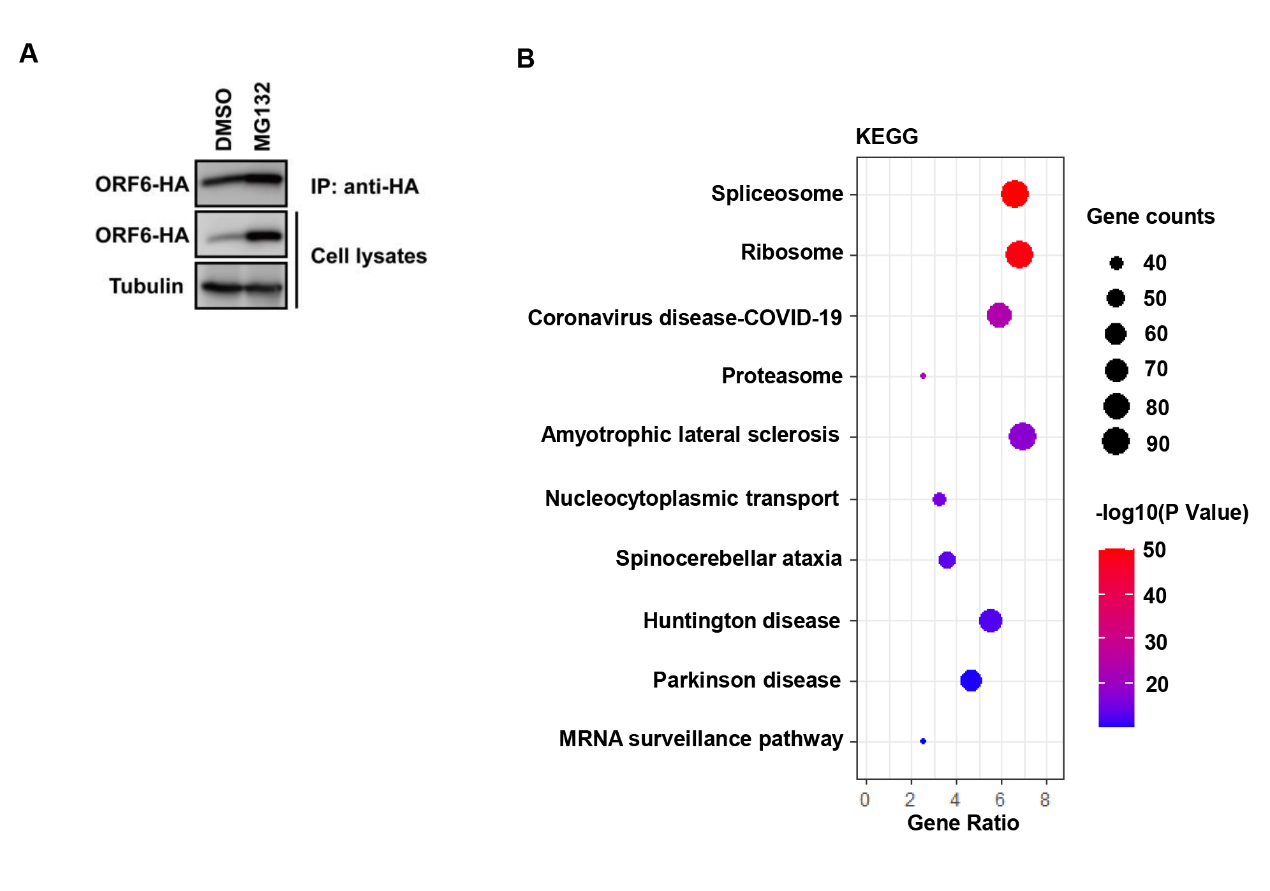


**Fig. S1. Ubiquitination-associated proteins interacted with ORF6. (A)** IP of the ORF6 protein after MG132 treatment. HEK293T cells transfected to express ORF6-HA were treated with MG132 for 10 h prior to harvest. Lysates were subjected to HA IP, and eluents were analyzed by IB. **(B)** Bubble plot of KEGG enrichment terms based on MS results. The alteration in color from red to blue illustrates a decrease in the *P*-value, while the size of the circles indicates the number of genes enriched in KEGG terms.


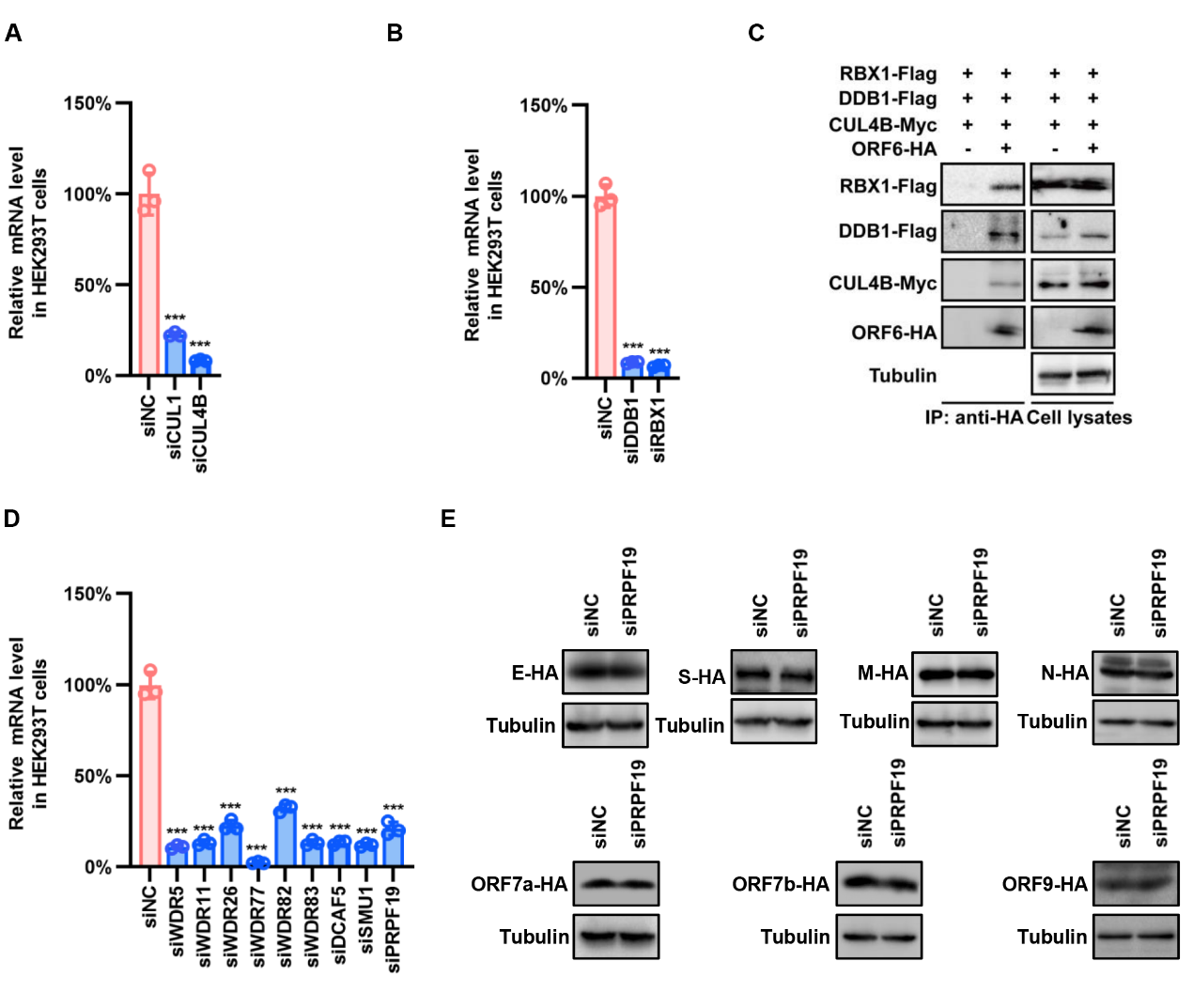


**Fig. S2. Identification of E3 ligase components involved in ORF6 degradation. (A-B, D)** The expression levels of the indicated factor in cells with corresponding factor knockdown were measured by qRT-PCR and normalized to GAPDH expression. **(C)** ORF6 interacts with CUL4B, RBX1 and DDB1. HEK293T cells transfected with constructs expressing ORF6-HA, CUL4B-Myc, RBX1-Flag, and DDB1-Flag were harvested and lysed. Lysates were subjected to HA antibody IP. The precipitates were analyzed by IB with the appropriate antibodies. **(E)** Knockdown of PRPF19 does not affect E, S, M, N, ORF7a, ORF7b, ORF9 stability. Data are representative of three independent experiments and shown as average ± SD (n = 3). Significance was determined by a two-tailed *t-*test: ****P* < 0.001.


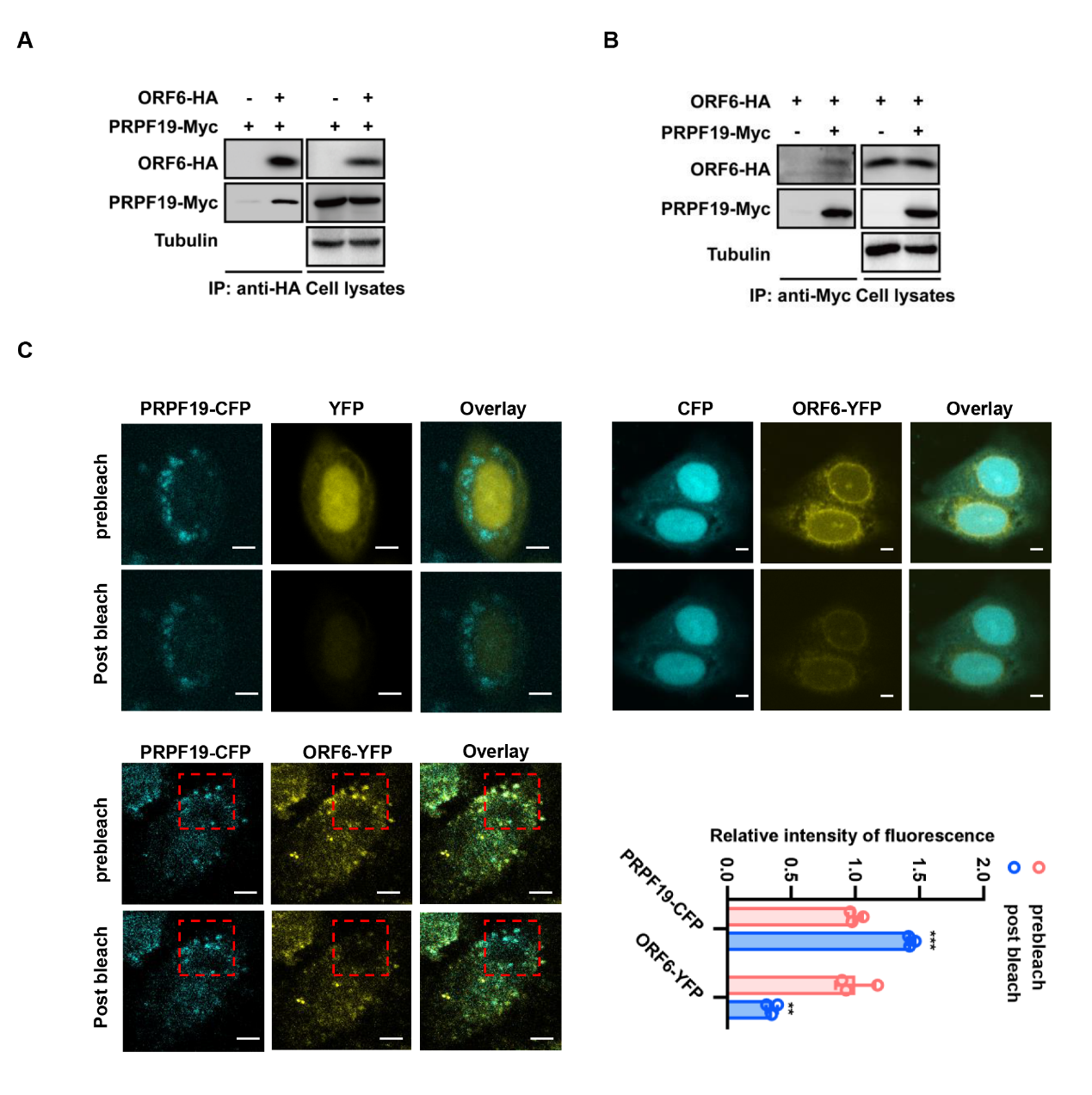


**Fig. S3. PRPF9 interacts with ORF6. (A-B)** HEK293T cells transfected with constructs expressing ORF6-HA and PRPF19-Myc were harvested and lysed. Lysates were subjected to Myc antibody IP or HA antibody IP. The precipitates were analyzed by IB with the appropriate antibodies. **(C)** Interaction between PRPF19 and ORF6 determined by FRET analysis. Hela cells transfected with ORF6-YFP and PRPF19-CFP were fixed. Then, a FRET assay was performed with a Zeiss LZM710 confocal microscope. Bars, 10 µm. The fluorescence intensity of images was analyzed by Image J.


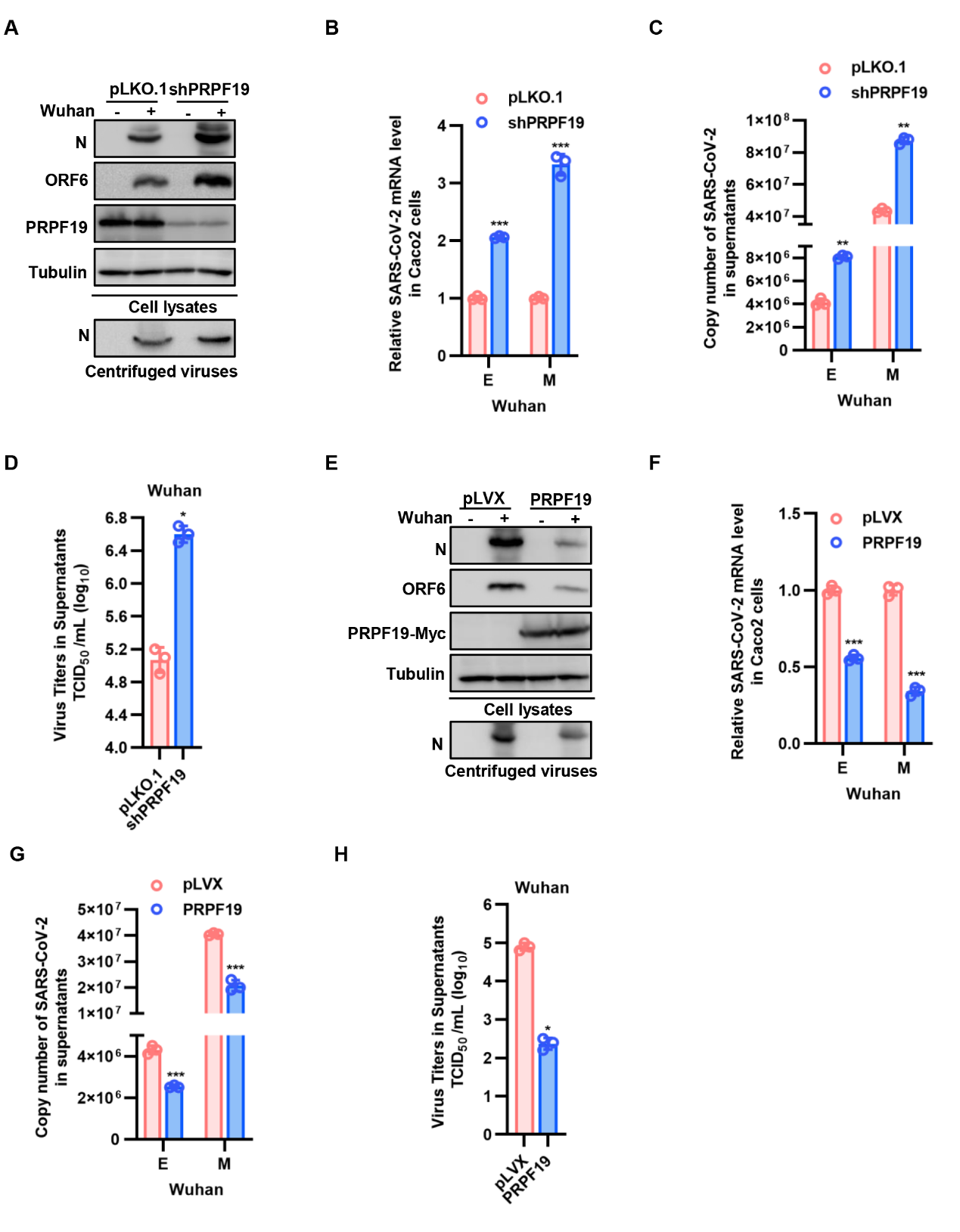


**Fig. S4. PRPF19 inhibits Wuhan strain replication.** PRPF19 knockdown in Caco2 cells increased Wuhan strain replication measured by determining the protein levels of ORF6 and N **(A)**, the mRNA levels of *E* and *M* genes in cells **(B)**, and in culture supernatants **(C)**, the virus titer in supernatants **(D)**. PRPF19 overexpression in Caco2 cells inhibited Wuhan strain replication measured by determining protein levels of ORF6 and N **(E)**, the mRNA levels of *E* and *M* genes in cells **(F)**, and in culture supernatants **(G)**, the virus titer in supernatants **(H)**. Data are representative of three independent experiments and shown as average ± SD (n = 3). Significance was determined by a two-tailed *t-*test: **P* < 0.05; ***P* < 0.01; ****P* < 0.001.


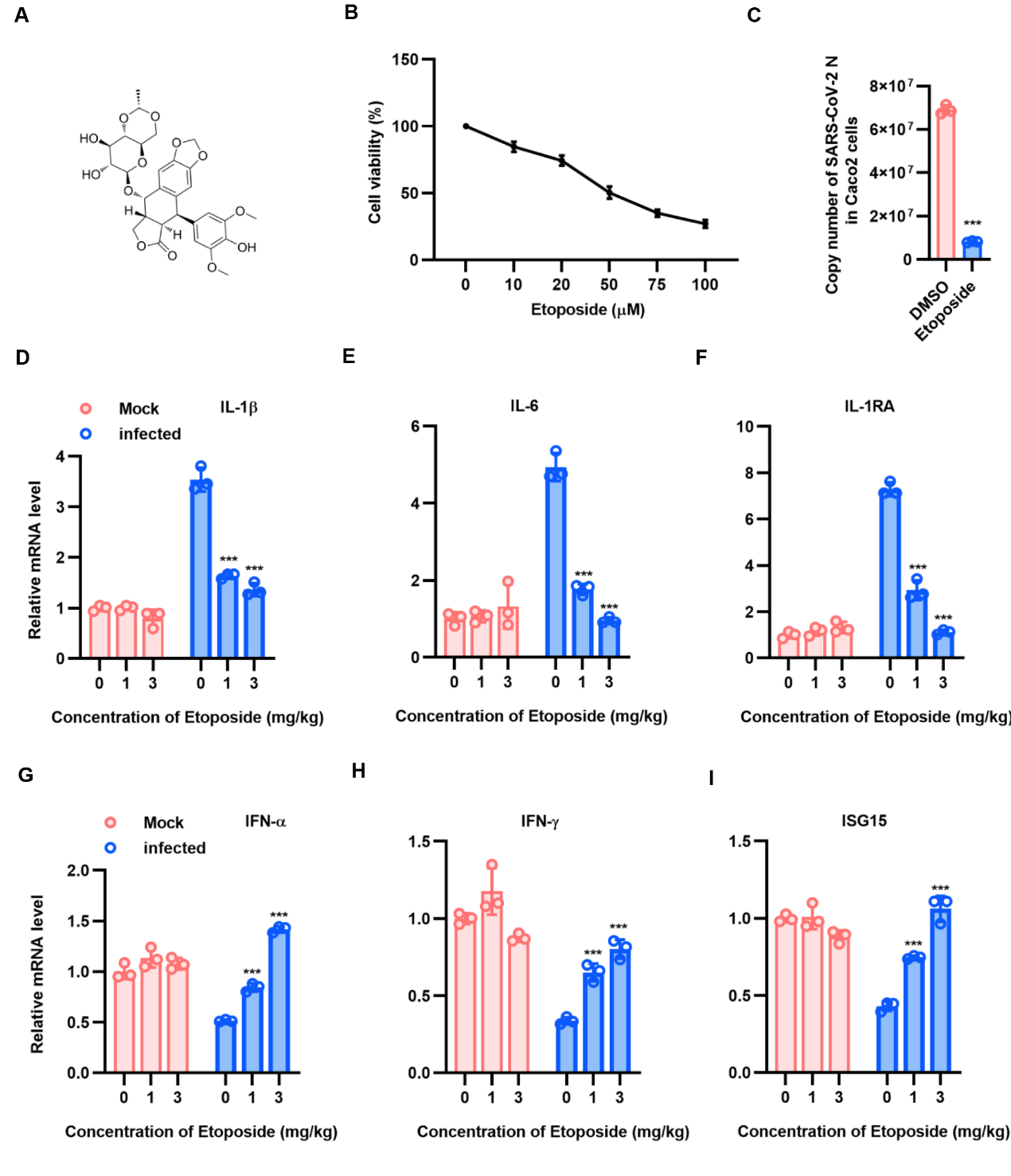


**Fig. S5. Etoposide alleviates the cytokine storm and IFN antagonism caused by SARS-CoV-2. (A)** The structural formula of Etoposide. **(B)** Cell viability of HEK293T induced by Etoposide after 12 h by CCK-8 assay. **(C)** Caco2 cells infected with SARS-CoV-2 were treated with or without Etoposide (5 μM) for 24 h. Viral replication was determined by the copy number of the *N* gene. **(D-I)** BLAB/C mice were treated with Etoposide at a dosage of 0, 1, or 3 mg/kg (20 g mouse, 30 μg, or 60 μg) four times at two-day intervals, then infected with the SARS-CoV-2 virus at a dosage of 10^5.5^ TCID_50_/ml via intraperitoneal injection. Spleens of each group were evaluated for the expression of cytokines and IFNs by RT-qPCR assay. B significance was determined by repeated measurement ANOVA, and C-H significance was determined by a two-tailed *t-*test. ****P* < 0.001.


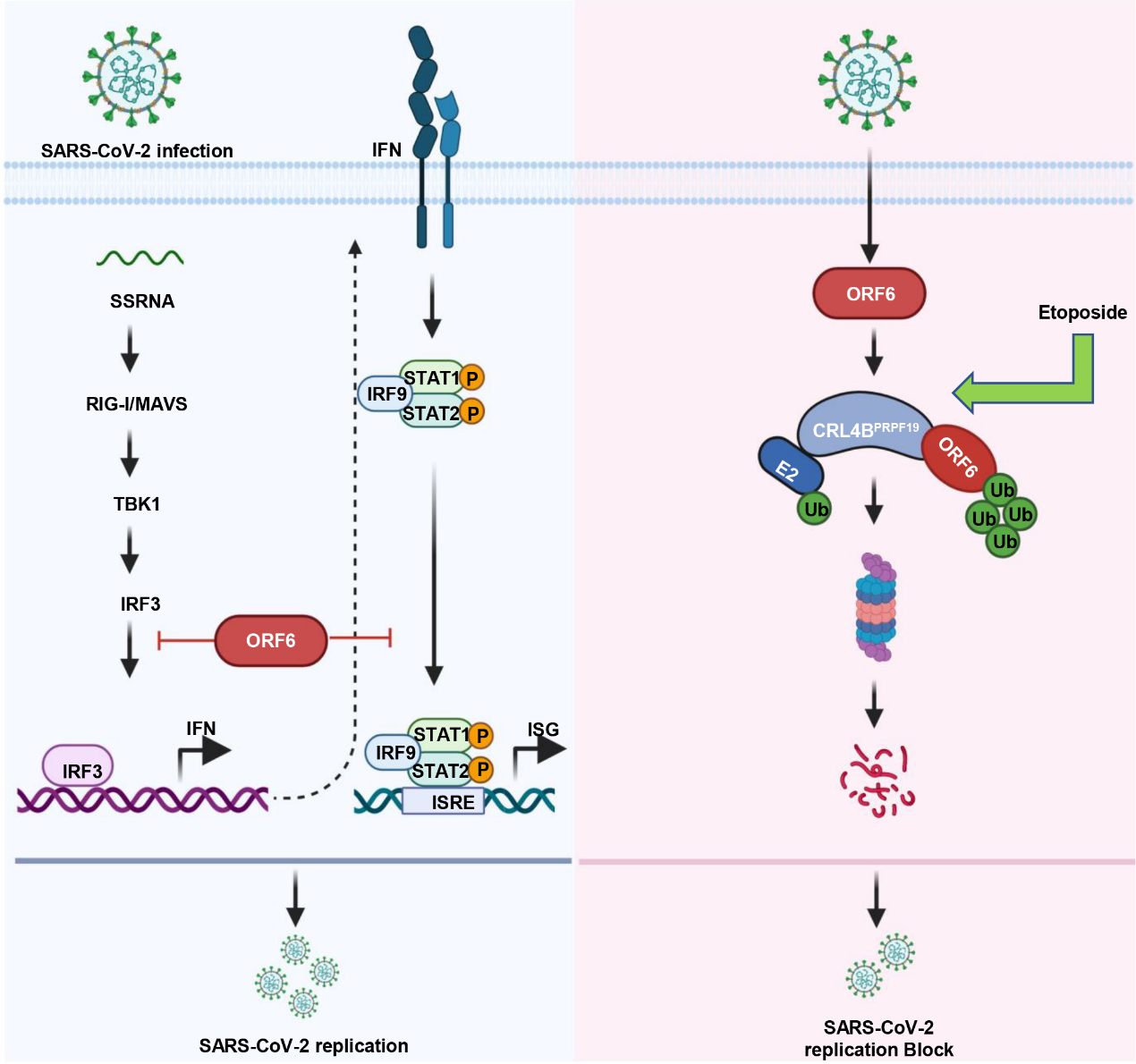


**Fig. S6. Graphical Abstract.** CRL4B^PRPF19^ mediates ORF6 ubiquitination and targets it for proteasomal degradation, further suppressing SARS-CoV-2 replication. The CUL4B pharmacological activator Etoposide promotes the ubiquitination process and inhibits viral replication.

**Table S1. Primers used in this study.**

| Primer | Sequence (5’-3’) | Enzyme site | Purpose |
| --- | --- | --- | --- |
| ORF6-K23R-F | TATTATGAGGACTTTTAGAGTTTCCATTTGGAATC | None | ORF6-K23R mutant |
| ORF6-K23R-R | CTAAAAGTCCTCATAATAATTAGTAATATC | None | ORF6-K23R mutant |
| ORF6-K38R-F | CATAAACCTCATAATTAGAAATTTATCTAAGTCAC | None | ORF6-K38R mutant |
| ORF6-K38R-R | CTAATTATGAGGTTTATGATGTAATCAAG | None | ORF6-K38R mutant |
| ORF6-K42R-F | ATAATTAAAAATTTATCTAGGTCACTAACTGAG | None | ORF6-K42R mutant |
| ORF6-K42R-R | CTAGATAAATTTTTAATTATGAGGTTTATG | None | ORF6-K42R mutant |
| ORF6-K48R-F | CACTAACTGAGAATAGATATTCTC | None | ORF6-K48R mutant |
| ORF6-K48R-R | CTATTCTCAGTTAGTGACTTAG | None | ORF6-K48R mutant |
| PRPF19-F | ATGTCCCTAATCTGCTCCATC | None | PRPF19-Myc-VR1012 |
| PRPF19-R | CTACAGGCTGTAGAACTTGAG | None | PRPF19-Myc-VR1012 |
| SalI-Myc-PRPF19-F | TGGGTCTTTTCTGCAGTCACCGTCGATGGAACAAAAACTCATATCAGAAGAAGATCTTATGTCCCTAATCTGCTCCATC | *SalI* | PRPF19-Myc-VR1012 |
| PRPF19-BglII-R | TGGCTGGCAACTAGAAGGCACAGCACTACAGAGAATAGAACTTGAGGCTTCTGTCCAT | *BglII* | PRPF19-Myc-VR1012 |
| XhoI-Myc-PRPF19-F | TCGCTAGCGCTACCGGACTCAGATCATGGAACAAAAACTCATATCAGAAGAAGATCTTATGTCCCTAATCTGCTCCATC | *XhoI* | PRPF19-Myc-pLVX |
| PRPF19-XbaI-R | CCGGTAGAATTATCTAGAGTCGCGGCTACAGGCTGTAGAACTTGAG | *XbaI* | PRPF19-Myc-pLVX |
| SalI-PRPF19-F | GAGCTCAAGCTTCGAATTCTGCAATGTCCCTAATCTGCTCCATC | *SalI* | PRPF19- pECFP |
| PRPF19-XbaI-R | TATGATCAGTTATCTAGATCCGGTGCTACAGGCTGTAGAACTTGAG | *BamHI* | PRPF19- pECFP |
| BamHI-ORF6-F | CGGGATCCATGTTTCATCTCGTTGACTTTC | *BamHI* | ORF6-YFP |
| ORF6-XhoI-R | CCCTCGAGATCAATCTCCATTGGTTGCTC | *XhoI* | ORF6-YFP |
| PRPF19-1-135-F | CTGGCTACCCTGAAATAACAGGCTGGC | None | PRPF19-1-135 truncation |
| PRPF19-1-135-R | TTTTCAGGGTAGCCAGAGCTTCTCGG | None | PRPF19-1-135 truncation |
| PRPF19-1-210-F | AAATACCGGCAGGTGTAATCCCACGTG | None | PRPF19-1-210 truncation |
| PRPF19-1-210-R | TCACCTGCCGGTATTTGCTGAGCTCT | None | PRPF19-1-210 truncation |
| SalI-Myc-PRPF19-WD-F | TGGGTCTTTTCTGCAGTCACCGTCGATGGAACAAAAACTCATATCAGAAGAAGATCTTATGGCAAGCATTCCTGGGATCCTG | *SalI* | PRPF19-WD truncation |
| PRPF19-WD-BglII-R | TGGCTGGCAACTAGAAGGCACAGCACTAGCTGTAGAACTTGAGGCTT | *BglII* | PRPF19-WD truncation |
| CUL1-RT-F | CAATGACGCTGGCTTTGTGGCT | None | Real-time qPCR |
| CUL1-RT-R | CAAGGAGTCACAGTATCGAGCC | None | Real-time qPCR |
| CUL4B-RT-F | GAAGCTACAGATGAAGAACTTGAG | None | Real-time qPCR |
| CUL4B-RT-R | GCACTCTTTCCGACTAACAGGC | None | Real-time qPCR |
| RBX1-RT-F | ACTGTGCCATCTGCAGGAACCA | None | Real-time qPCR |
| RBX1-RT-R | ACCTGTCGTGTTTTGAGCCAGC | None | Real-time qPCR |
| DDB1-RT-F | CATTCCTCGCTCCATCCTGATG | None | Real-time qPCR |
| DDB1-RT-R | CCTTCTTACGGTCGCTCAACAG | None | Real-time qPCR |
| WDR5-RT-F | AGTGCCTCAAGACTTTGCCAGC | None | Real-time qPCR |
| WDR5-RT-R | CGATGAGCGTCTTCAGGCACTG | None | Real-time qPCR |
| WDR11-RT-F | AGACAGTGCTCGGATTCCACCA | None | Real-time qPCR |
| WDR11-RT-R | CTGGATACTCTGCCTTTCAAGTC | None | Real-time qPCR |
| WDR26-RT-F | GTGGCTTGGAATCCAGATGGGA | None | Real-time qPCR |
| WDR26-RT-R | CCATCACTCAAGCACCAAAGGC | None | Real-time qPCR |
| WDR77-RT-F | CTCAGGTCACTTGTGTTGCTGC | None | Real-time qPCR |
| WDR77-RT-R | ATCTGTGATGCTGGCTTGGGAC | None | Real-time qPCR |
| WDR82-RT-F | TCCTCATTTCCACCAACGGCAG | None | Real-time qPCR |
| WDR82-RT-R | ATGAAGCCTCCAGTGTGACAGC | None | Real-time qPCR |
| WDR83-RT-F | GCAGTGACAAGACGCTGAAGCT | None | Real-time qPCR |
| WDR83-RT-R | CGGAGCAGAGACTACTGTTGTC | None | Real-time qPCR |
| DCAF5-RT-F | AACGGAGCCTTCATGGTGCTGA | None | Real-time qPCR |
| DCAF5-RT-R | CCAGTACATCCTGGCTGCTTGT | None | Real-time qPCR |
| SMU1-RT-F | TTAGCTGGCGAAGTCAGTGTGG | None | Real-time qPCR |
| SMU1-RT-R | GTCATACCAGGAGGAAGCAATCC | None | Real-time qPCR |
| PRPF19-RT-F | TGGGCTTTCTCTGACATCCAGAC | None | Real-time qPCR |
| PRPF19-RT-F | CCTGTTCCAAAGATGAGTCCGTC | None | Real-time qPCR |
| IFN-α-RT-F | AGAAGGCTCCAGCCATCTCTGT | None | Real-time qPCR |
| IFN-α-RT-R | TGCTGGTAGAGTTCGGTGCAGA | None | Real-time qPCR |
| IFN-β-RT-F | CTTGGATTCCTACAAAGAAGCAGC | None | Real-time qPCR |
| IFN-β-RT-R | TCCTCCTTCTGGAACTGCTGCA | None | Real-time qPCR |
| IFIT1-RT-F | GCCTTGCTGAAGTGTGGAGGAA | None | Real-time qPCR |
| IFIT1-RT-R | ATCCAGGCGATAGGCAGAGATC | None | Real-time qPCR |
| IFIT3-RT-F | CCTGGAATGCTTACGGCAAGCT | None | Real-time qPCR |
| IFIT3-RT-R | GAGCATCTGAGAGTCTGCCCAA | None | Real-time qPCR |
| ISG15-RT-F | CTCTGAGCATCCTGGTGAGGAA | None | Real-time qPCR |
| ISG15-RT-R | AAGGTCAGCCAGAACAGGTCGT | None | Real-time qPCR |
| GAPDH-RT-F | CCCATCACCATCTTCCAGG | None | Real-time qPCR |
| GAPDH-RT-R | TTCTCCATGGTGGTGAAGAC | None | Real-time qPCR |
| E-SARS-CoV-2-RT-F | CGATCTCTTGTAGATCTGTTCTC | None | Real-time qPCR |
| E-SARS-CoV-2-RT-R | ATATTGCATTGCAGCAGTACGCACA | None | Real-time qPCR |
| M-SARS-CoV-2-RT-F | GGTTTCCTATTCCTTACATGG | None | Real-time qPCR |
| M-SARS-CoV-2-RT-R | ATTCTGTAAACAGCAGCAAGC | None | Real-time qPCR |
| Mus-IL-1B-RT-F | TGTGGAGAAGCTGTGGCAG | None | Real-time qPCR |
| Mus-IL-1B-RT-R | CAGCAGGTTATCATCATCATC | None | Real-time qPCR |
| Mus-IL-6-RT-F | CACTTCACAAGTCGGAGGC | None | Real-time qPCR |
| Mus-IL-6-RT-R | TTTGTATCTCTGGAAGTTTCAG | None | Real-time qPCR |
| Mus-IL-1RA-RT-F | ACCTGAGAAACAACCAGCTC | None | Real-time qPCR |
| Mus-IL-1RA-RT-R | CTTGGCACAAGACAGGCAC | None | Real-time qPCR |
| Mus-IFN-α-RT-F | GGATGTGACCTTCCTCAGACTC | None | Real-time qPCR |
| Mus-IFN-α-RT-R | ACCTTCTCCTGCGGGAATCCAA | None | Real-time qPCR |
| Mus-IFN-γ-RT-F | CAGCAACAGCAAGGCGAAAAAGG | None | Real-time qPCR |
| Mus-IFN-γ-RT-R | TTTCCGCTTCCTGAGGCTGGAT | None | Real-time qPCR |
| Mus-ISG15-RT-F | CATCCTGGTGAGGAACGAAAGG | None | Real-time qPCR |
| Mus-ISG15-RT-R | CTCAGCCAGAACTGGTCTTCGT | None | Real-time qPCR |
| Mus-GAPDH-RT-F | CAAGGCCGAGAATGGGAAG | None | Real-time qPCR |
| Mus-GAPDH-RT-R | TCCATGGTGGTGAAGACAC | None | Real-time qPCR |
